# An exact algorithm for finding cancer driver somatic genome alterations: the weighted mutually exclusive maximum set cover problem

**DOI:** 10.1186/s13015-016-0073-9

**Published:** 2016-05-04

**Authors:** Songjian Lu, Gunasheil Mandava, Gaibo Yan, Xinghua Lu

**Affiliations:** Department of Biomedical Informatics, University of Pittsburgh, Pittsburgh, PA 15206 USA

**Keywords:** Mutual exclusivity, Gene signature, Somatic genome alteration

## Abstract

**Background:**

The mutual exclusivity of somatic genome alterations (SGAs), such as somatic mutations and copy number alterations, is an important observation of tumors and is widely used to search for cancer signaling pathways or SGAs related to tumor development. However, one problem with current methods that use mutual exclusivity is that they are not signal-based; another problem is that they use heuristic algorithms to handle the NP-hard problems, which cannot guarantee to find the optimal solutions of their models.

**Method:**

In this study, we propose a novel signal-based method that utilizes the intrinsic relationship between SGAs on signaling pathways and expression changes of downstream genes regulated by pathways to identify cancer signaling pathways using the mutually exclusive property. We also present a relatively efficient exact algorithm that can guarantee to obtain the optimal solution of the new computational model.

**Results:**

We have applied our new model and exact algorithm to the breast cancer data. The results reveal that our new approach increases the capability of finding better solutions in the application of cancer research. Our new exact algorithm has a time complexity of $O^{*}(1.325^{m})$(**Note:** Following the recent convention, we use a star * to represent that the polynomial part of the time complexity is neglected), which has solved the NP-hard problem of our model efficiently.

**Conclusion:**

Our new method and algorithm can discover the true causes behind the phenotypes, such as what SGA events lead to abnormality of the cell cycle or make the cell metastasis lose control in tumors; thus, it identifies the target candidates for precision (or target) therapeutics.

**Electronic supplementary material:**

The online version of this article (doi:10.1186/s13015-016-0073-9) contains supplementary material, which is available to authorized users.

## Background

Cancers are genomic diseases in which genomic perturbations, such as mutations or copy number alterations of genes encoding signal proteins, lead to perturbed cellular signal pathways, which in turn causes uncontrolled cell growth. An important area of cancer research is to discover perturbed signal transduction pathways in cancers in order to gain insight into disease mechanisms and guide patient treatment. Since contemporary biotechnologies can easily detect SGAs in tumor cells, including mutations and copy number alterations, a huge amount of SGA data is readily available. For example, The Cancer Genome Atlas (TCGA) contains somatic mutation, copy number alteration, and gene expression data for more than ten thousand cancer samples. All of these data give us an unprecedented opportunity to study cancer signaling pathways. However, each tumor usually has up to hundreds of SGAs, and they disperse in different pathways, some driving tumor genesis and others not related to cancers at all. Moreover, the SGA event that perturbs a particular pathway in one tumor may be on a different gene to the SGA event that perturbs the same pathway in another tumor. Hence, it is a challenge to find SGAs across different patients that affect a common cancer signaling pathway.

Mutual exclusivity is an important property that is helpful in searching for driver SGAs, i.e., SGAs that lead to tumor development. This property is based on the observation that SGA events among the genes constituting a signaling pathway tend to occur in a mutually exclusive fashion [1, 2], i.e., each tumor usually does not have two or more SGA events affecting the same pathway. This is because SGAs are relatively rare events, and one mutation or copy number alteration in such a pathway is usually sufficient to disrupt the signal that prevents cancers.

Although mutual exclusivity has been successfully applied in searching for driver SGAs [3–6], the methods used in previous research to find driver SGAs have some limitations. One limitation is that all previous methods applied mutual exclusivity to all tumors in the given data set rather than applied this property to a selected subset of tumors with SGAs that perturbed a common signal. In their solutions, a set of genes with mutually exclusive SGA events could easily come from multiple pathways, including pathways that are not related to tumor development, as SGA events of genes in the same pathway having the mutually exclusive tendency does not mean that genes with a mutually exclusive SGA pattern are in the same signaling pathway. Another problem is that previous works used heuristic algorithms when the computational problems of their models were NP-hard [5, 6], which cannot guarantee to find the optimal solutions of their models.

In this paper, we first propose an improved model that can increase the possibility that genes of a solution with mutually exclusive SGA events come from a common pathway related to tumor development. We first compared the expressions of a gene signature in tumor and normal cells and used this information to partition the tumors under investigation into two subsets: those with an abnormal and a normal expression of the gene signature. As genes in a gene signature are usually co-expressed and the expression pattern of a gene signature decides biological phenotype or medical condition of cells, it is highly likely that the expression of a gene signature is regulated by a common pathway and the abnormal expressions of the signature in a subset of tumors are due to SGA events that perturb the common signal that regulates the signature. The partition of tumors was also used to evaluate the associations of SGA events to the common signal, i.e., to compute the information of SGA events with respect to a common signal. We then applied mutual exclusivity only to the subset of tumors with abnormal expressions of the gene signature (exclude tumors with normal expressions of the gene signature) and considered the information of SGAs with respect to the common signal that regulated the gene signature. Hence, our new method is signal-based. We also presented an exact algorithm to solve the NP-hard computational problem in our model, which guaranteed to obtain the optimal solutions of our model. We successfully applied this exact algorithm to search for driver SGAs in a previous research [7]. In this new paper, we give detail of the algorithm, the proof of its correctness and time complexity, and a new application of the algorithm in breast cancer data.

In the next two sections, we define the computational problem of our model and present an efficient exact algorithm to solve it. In the section Data and Methods, we present how to transform the biological problem into our computational problem.

## The weighted mutually exclusive maximum set cover problem is NP-hard

In this paper, we formulate the driver SGA finding problem into a weighted mutually exclusive maximum set cover problem. The formal definition of the problem is: given a ground set *X* of *n* elements, a collection $\mathcal{F}$ of *m* subsets of *X*, and a weight function $w: \mathcal{F} \rightarrow (-\infty , \infty )$, if $\mathcal{F'} =\{S_1,S_2,\ldots ,S_h\} \subset \mathcal{F}$ such that $|(\cup _{i=1}^hS_i)|$ is maximized, and $S_i\cap S_j=\emptyset$ for any $i \ne j$, then we say $\mathcal{F'}$ is a mutually exclusive maximum set cover of *X* and $\sum _{i=1}^hw(S_i)$ is the weight of $\mathcal{F'}$; the goal of the problem is to find the mutually exclusive maximum set cover of *X* with the minimum weight, i.e., if there is more than one mutually exclusive maximum set cover of *X*, we choose the one with the minimum weight.

In this section, we first prove that the mutually exclusive maximum set cover problem, i.e. all subsets in $\mathcal{F}$ have weight 0, is NP-hard, which would in turn prove that the weighted mutually exclusive maximum set cover problem is NP-hard.

We will prove the NP-hardness of the mutually exclusive maximum set cover problem by reducing another NP-hard problem, the maximum 3-set packing problem, to it. Recall that the maximum 3-set packing problem is: given a collection $\mathcal{S}$ of subsets, where the size of each subset in $\mathcal{S}$ is three, try to find an $\mathcal{S}' \subset \mathcal{S}$ such that subsets in $\mathcal{S}'$ are pairwise disjoint and $|\mathcal{S}'|$ is maximized.

### **Theorem 1**

*The mutually exclusive maximum set cover problem is NP-hard.*

### *Proof*

Let $\mathcal{S} = \{S_1,S_2,\ldots ,S_m\}$ be an instance of the maximum 3-set packing problem. We create an instance of the mutually exclusive maximum set cover problem such that $X = \cup _{i=1}^mS_i$ and $\mathcal{F} = \mathcal{S}$.

It is obvious that $\mathcal{P} = \{P_1,P_2,\ldots , P_k\}$ is a solution of the mutually exclusive maximum set cover problem if and only if $\mathcal{P} = \{P_1,P_2,\ldots , P_k\}$ is a solution of the maximum 3-set packing problem. Thus, the mutually exclusive maximum set cover problem is NP-hard. $\square$

Since the mutually exclusive maximum set cover problem is a special case of the weighted mutually exclusive maximum set cover problem, the Theorem 1 implies that the weighted mutually exclusive maximum set cover problem is NP-hard. In the Data and Methods section, we will introduce in detail how to formulate a biological problem into a weighted mutually exclusive maximum set cover problem. Next, we introduce background related to this problem.

The research on weighted mutually exclusive maximum set cover problems is limited. To the best of our knowledge, only Björklund et al. [8] give an algorithm of $O^*(2^n)$ for the related problem of finding *k* mutually exclusive subsets $\mathcal{S} = \{S_1,S_2,\ldots , S_k\}$ such that $\mathcal{S}$ has the maximum weight sum and covers all elements in *X* (the solution may not exist). The weighted mutually exclusive maximum set cover problem is closely related to the set cover problem, which is a well-known NP-hard problem in Karp’s 21 NP-complete problems [9]. Much research about the set cover problem has been focused on approximation algorithms, such as papers  [10–13] giving polynomial time approximation algorithms that find solutions whose sizes are at most $c\log n$ times the size of the optimal solution, where *c* is a constant. There is also plenty of research about the hitting set problem, which is equivalent to the set cover problem. In this direction of research, people have mainly designed fixed-parameter tractable (FPT) algorithms that use the solution size *k* as a parameter for the hitting set problem under the constraint that the sizes of all subsets in the problem are bounded by *d*. For example, Niedermeier et al. [14] gave a $O^*(2.270^k)$ algorithm, and Fernau et al. [15] gave a $O^*(2.179^k)$ algorithm for the 3-hitting set problem, respectively. Very recently, people have also studied an extended version of the set cover problem that finds a subset $\mathcal{F'}$ of $\mathcal{F}$ such that each element in *X* is covered by at least *t* subsets in $\mathcal{F'}$. For example, Hau et al. [16] designed an algorithm with time complexities of $O^*((t+1)^n)$ for the problem; Lu et al. [17] further improved the algorithm under the constraint that some elements in *X* are included in at most *d* subsets in $\mathcal{F}$. These two algorithms can be easily modified to solve the weighted mutually exclusive maximum set cover problem. However, in the application of this work, *n*, the number of tumor samples, is large (can be several hundreds). Therefore, use of these two algorithms is not practical. At the same time, in this study, the number of genes with SGAs is bounded by a small number that is much smaller than the number of tumors. Hence, there is a need to design better algorithms solving the weighted mutually exclusive maximum set cover problem, ones that use *m*, the number of subsets (genes with SGAs) in $\mathcal{F}$, as parameter. We offer such an algorithm bellow.

## The main algorithm

In this section, we introduce our main algorithm. The basic idea of our method is branch and bound. The algorithm first finds a subset in $\mathcal{F}$ and then branches on it. By the mutual exclusivity principle, if any two subsets in $\mathcal{F}$ overlap, then at most one of them can be chosen into the solution. Hence, suppose that the subset *S* intersects with other *d* subsets in $\mathcal{F}$; then if *S* is included into the solution, *S* and the other *d* subsets intersecting with *S* will be removed from the problem, and if *S* is excluded from the solution, *S* will be removed from the problem. We continue this process until the resulting sub-problems can be solved in constant or polynomial time. Letting *T*(*m*) be the number of leaves of the search tree when calling the algorithm with *m* subsets in $\mathcal{F}$, we can obtain the recurrence relation $T(m) \le T(m -(d+1)) + T(m-1)$. As when $d=0$, the subset *S* does not have a nonempty intersection with all other subsets in $\mathcal{F}$, we do not need to branch and include *S* into the solution directly. As a result, we can suppose that $d\ge 1$. It is easy to verify that if $d\ge 1$, $T(m) \le 1.619^m$ satisfies this recurrence relation. In this paper, we improve the running time to solve the problem by carefully selecting subsets in $\mathcal{F}$ for branching. The execution process of the algorithm involves going through a search tree and the running of the algorithm is proportional to the number of leaves in the search tree.

Before presenting our major results, we prove three lemmas. Given an instance $(X, \mathcal{F}, w)$ of the weighted mutually exclusive maximum set cover problem, we make a graph *G*, called the intersection graph, such that each subset in $\mathcal{F}$ makes a node in *G*, and if any two subsets have a nonempty intersection, then an edge is added between them. For convenience, in the rest of paper, we use “a node in the intersection graph” and “a subset in $\mathcal{F}$” interchangeably.

Suppose $C=(V_c, E_c)$ is a connected component of *G*. We denote $(X,\mathcal{F},w)_C$ the sub-instance induced by component *C*, i.e., $(X,\mathcal{F},w)_C = (\cup _{S\in V_c}S,V_c,w)$. In the algorithm, we say $Solution_1$ is better than $Solution_2$ if 1) $Solution_1$ covers more elements in *X* than $Solution_2$ covers, or 2) $Solution_1$ and $Solution_2$ cover the same number of elements in *x*, but the weight of $Solution_1$ is less than the weight of $Solution_2$. In the intersection graph, *neighbor*(*S*) includes *S* and all nodes that are connected to *S*.

The first lemma shows that we can find the solution of the problem by finding the solutions of all sub-instances induced by connected components of the intersection graph *G*.

### **Lemma 1**

*Given an instance *$(X, \mathcal{F}, w)$* of the weighted mutually exclusive maximum set cover problem, if the intersection graph obtained from the instance consists of several connected components, then the solution of the problem is the union of solutions of all sub-instances induced by the connected components.*

### *Proof*

As the subsets in each sub-instance have no elements in other sub-instances, we can solve each sub-instance independently. It is obvious that the optimal solutions of all sub-instances make the optimal solution of the original instance. $\square$

**Fig. 1:**
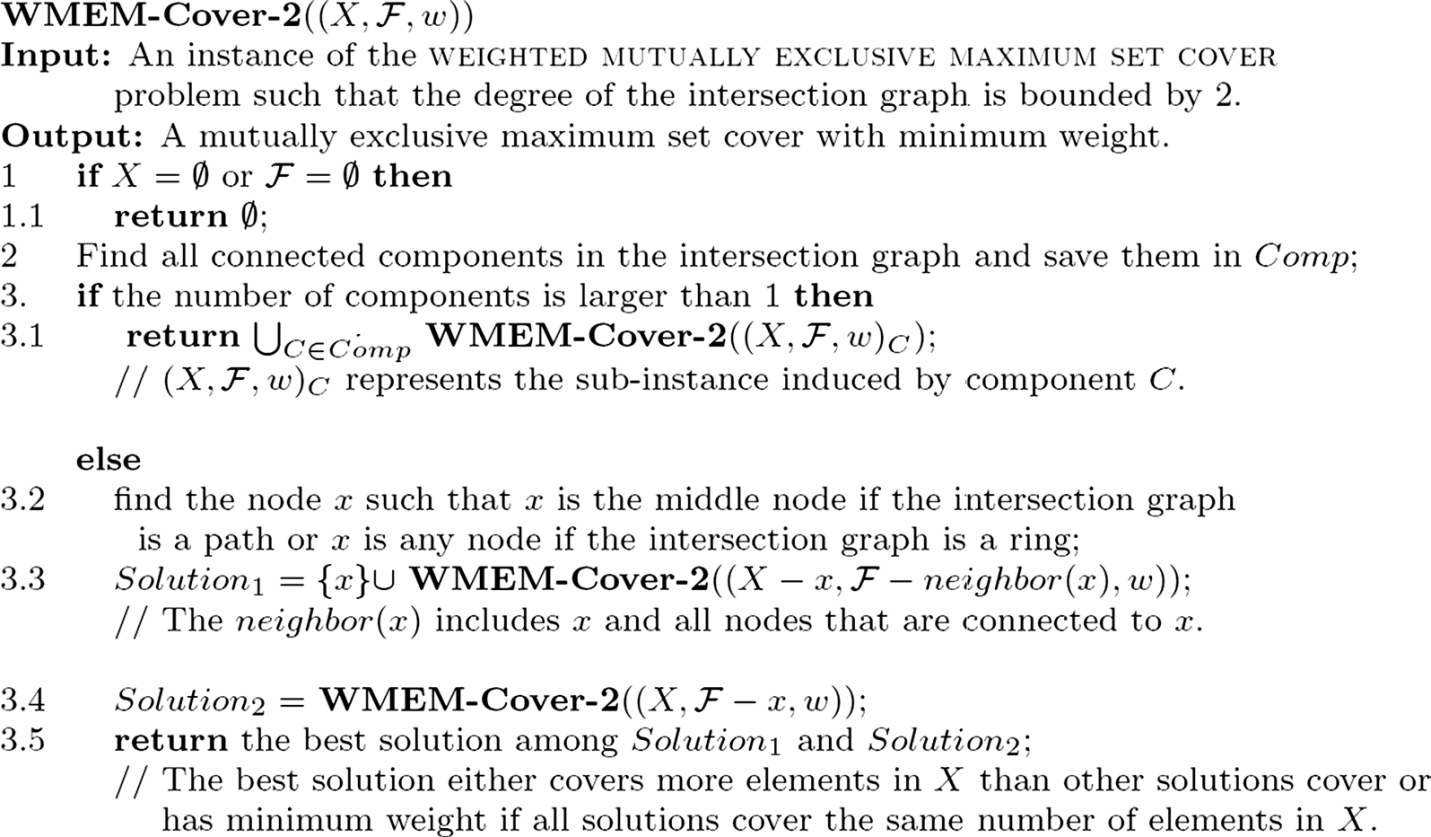
Algorithm for the weighted mutually exclusive maximum set cover problem with overlapped degrees bounded by two

In the next lemma, we show that if the maximum degree of the intersection graph obtained from the given instances is bounded by two, i.e., each subset in the instance is overlapped with at most two other subsets, then the problem can be solved in polynomial time (Fig. 1).

### **Lemma 2**

*Given an instance*$(X, \mathcal{F}, w)$ of the *weighted mutually exclusive maximum set cover problem, if the degree of its intersection graph is bounded by two, then the problem can be solved in*$O(m^2)$* time.*

### *Proof*

We first prove that if the intersection graph has only one connected component, the running time of the algorithm **WMEM-Cover-2** is polynomial (refer to Fig. 1).

As the degree of the intersection graph is bounded by two, the connected component can only be a simple path or a simple ring.

Case 1: Suppose that the intersection graph is a simple path. The algorithm first finds the middle node (subset) *x* of the path (Note: when the path has even nodes, choose any one of the two middle nodes.); then it branches on *x* such that branch one includes the node into the solution (three subsets are removed from the problem) and branch two excludes the node from the solution (one subset is removed from the problem). After the branching, the resulting intersection graphs are two connected components of almost equal sizes. Hence, if *T*(*m*) represents the number of leaves in the search tree, we have

$$\begin{aligned} T(m)\le (T(\lceil (m-3)/2\rceil ) + T(\lfloor (m-3)/2\rfloor )) + (T(\lceil (m-1)/2\rceil ) + T(\lfloor (m-1)/2\rfloor )) < 4T(m/2). \end{aligned}$$

From this recurrence relation, we have

$$\begin{aligned} T(m) \le 4^{\log m} = m^2. \end{aligned}$$

Case 2: Suppose that the intersection graph is a simple ring. The algorithm chooses any node and branches on it. Similar to case 1, one branch removes three subsets from the problem while the other branch removes one subset from the problem. After this operation, the resulting intersection graphs in both branches are simple paths. So with the analysis of case 1, we can obtain

$$\begin{aligned} T(m) \le (m-3)^2 + (m-1)^2 < 2m^2. \end{aligned}$$

If the intersection graph of the instance has multiple connected components, then by Lemma 1, we can solve sub-instances induced by connected components independently. As each sub-instance induced by a connected component can be solved in polynomial time, the original instance can be solved in polynomial time. It is easy, then, to obtain that the running time is bounded by $O(m^2)$.

The correctness of the algorithm is straightforward. The algorithm **WMEM-Cover-2** first chooses a node in the intersection graph, then branches on it. One branch includes the node into the solution while the other branch excludes the node from the solution. Hence, all possible combinations of mutually exclusive covers are considered and the algorithm returns the best solution, i.e., the solution that covers the maximum number of elements in *X* and has the minimum weight. $\square$

As an anonymous reviewer suggested, if the degree of nodes in the intersection graph is bounded by 2, then the problem can be solved by a dynamic programming algorithm with a time complexity of *O*(*m*). The basic idea is that if $v_1, v_2, ..., v_k$ make a simple path and *f*(*i*) represents the current best sub-solution chosen from $v_1, v_2, ..., v_i$, then $f(i+1)$ can be obtained from $f(i-1)$, $v_{i+1}$ and *f*(*i*). We have, however, decided to leave this for future work.

**Fig. 2:**
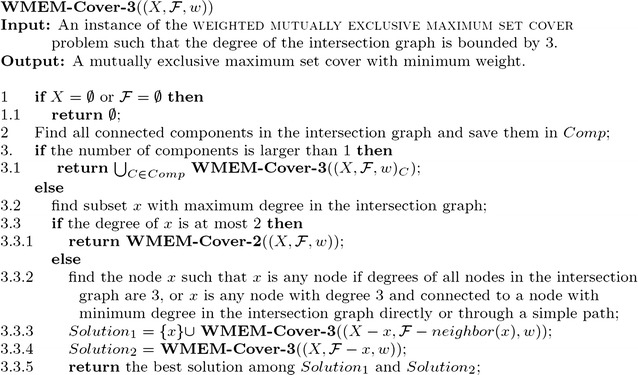
The main algorithm for the weighted mutually exclusive maximum set cover problem with overlapped degrees bounded by three

In the next lemma, we present how to improve the running time of the algorithm when the degree of nodes in the intersection graph is bounded by three (Fig. 2).

### **Lemma 3**

*Given an instance *$(X, \mathcal{F}, w)$ of the *weighted mutually exclusive maximum set cover problem, if the degree of its intersection graph is bounded by three, then the problem can be solved in *$O^{*}(1.325^{m})$^1^* time.*

### *Proof*

We suppose that the intersection graph always has a node whose degree is less than three. At the beginning, if all nodes in the intersection graph have degree three, then after the first branching, both subgraphs will have at least three nodes whose degrees are at most two. After that, when the algorithm makes new branchings, it is obvious that there are always new nodes whose degrees will be reduced. Hence, after the first branching, the intersection graph will always keep at least one node of degree bounded by two.

The algorithm **WMEM-Cover-3**  (refer to Fig. 2) first always finds a node *x* of degree three that is connected to a node with minimum degree (less than three) in the intersection graph directly or through a simple path, then branches at *x*. We analyze the running time of the algorithm **WMEM-Cover-3** by considering the following cases.

**Fig. 3:**
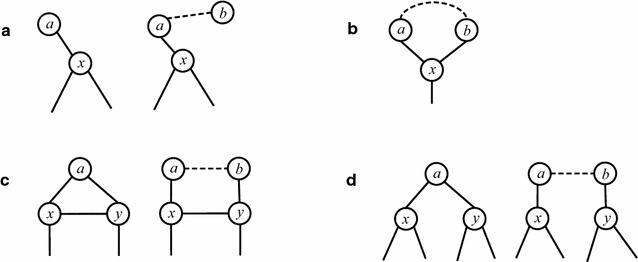
Different structures in the intersection graph with degree bounded by three. The dashed line means that nodes *a* and *b* are connected by an edge or a simple path

**Case 1.** The node *x* is connected by a simple path *P* such that one end of the path has degree one (refer to Fig. 3a). In the branch including *x* into the solution, *x* and three neighbors of *x* are removed. In the branch that excludes *x* from the solution, *x* is removed; the simple path *P* becomes an isolated component and the sub-instance induced by *P* can be solved in polynomial time; thus at least two nodes are removed in this branch. We obtain the recurrence relation

$$\begin{aligned} T(m)\le T(m-4) + T(m-2), \end{aligned}$$

which leads to $T(m)\le 1.273^m$

.

**Case 2.** Both ends of the simple path *P* are connected to *x* (refer to Fig. 3b), where in this case, the length of the simple path *P* is at least two. Then in the branch including *x* into the solution, as with case 1, at least four nodes are removed and in the branch excluding *x* from the solution, the path *P* also becomes an isolated component. Hence, we have

$$\begin{aligned} T(m)\le T(m-4) + T(m-3), \end{aligned}$$

which leads to $T(m)\le 1.221^m$.

**Case 3.** One end of the simple path *P* is connected to *x* while the other end of *P* is connected to node *y* that is not *x*, where *x* and *y* can be connected by an edge or not (refer to Fig. 3c, d). In the branch that includes *x* into the solution, as with the above cases, at least four nodes are removed. In the other branch, after *x* is removed, a node of degree one is generated. If no node(s) of degree one is(are) in the connected component with nodes of degree three, then the node(s) of degree one is(are) in connected component(s) bounded by two. Hence, we have

$$\begin{aligned} T(m)\le T(m-4) + T(m-2), \end{aligned}$$

which leads to $T(m)\le 1.273^m$. If there is at least one node of degree one in the connected component with nodes of degree three, then the next branching is as in Case 1 (with node *x* removed). Therefore, even in the worst case, we have the recurrence relation

$$\begin{aligned} T(m) \le T(m-4) + (T(m-5) + T(m-3)), \end{aligned}$$

which leads to $T(m)\le 1.325^m$.

The above analysis has included all possible situations where a node of degree at most two is connected to a node of degree three. Hence, we can obtain that the time complexity of the algorithm is $O^*(1.325^m)$.

As with Lemma 2, the correctness of the algorithm **WMEM-Cover-3** is obvious. $\square$

Next, we present the main results.

### **Theorem 2**

*The weighted mutually exclusive maximum set cover problem can be solved in*$O^*(1.325^m)$* time.*

### *Proof*

**Fig. 4:**
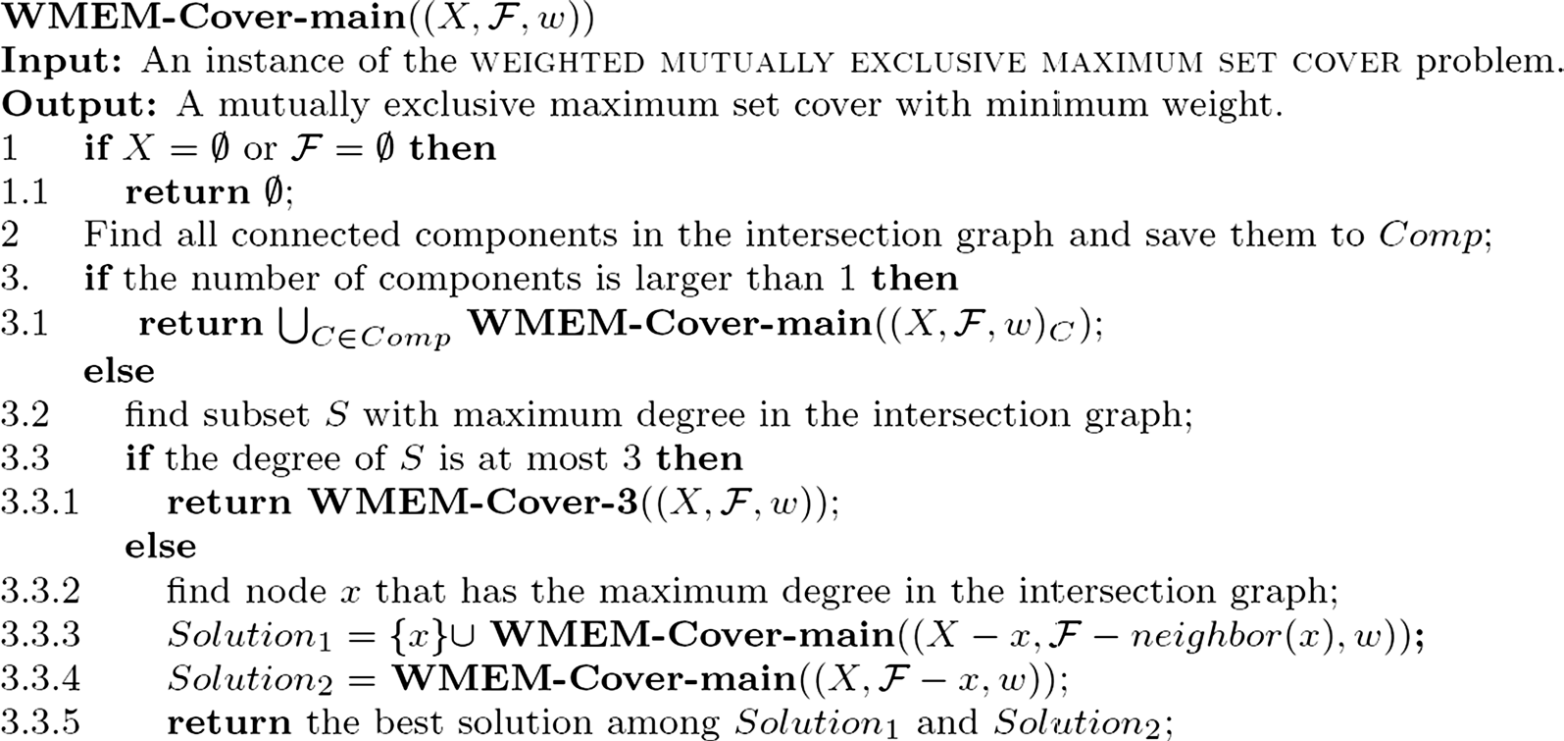
The main algorithm for the weighted mutually exclusive maximum set cover problem

Like the above lemmas, the correctness of the algorithm **WMEM-Cover-main** is trivial (refer to Fig. 4) . We only prove the running time of the algorithm.

The algorithm **WMEM-Cover-main** always keeps searching for the node *x* with the maximum degree in the intersection graph; then it branches on the node. If the degree of *x* is *d*, then we obtain the recurrence relation

$$\begin{aligned} T(m) \le T(m-(d+1)) + T(m-1). \end{aligned}$$

Furthermore, if $d\le 3$, the algorithm **WMEM-Cover-main** will call the algorithm **WMEM-Cover-3**. Hence, we always have $d \ge 4$ when we use algorithm **WMEM-Cover-main** to branch, which leads to $T(m)\le 1.325^m$. From Lemma 3, if $d\le 3$, we also have $T(m)\le 1.325^m$. Therefore, the overall running time of the algorithm **WMEM-Cover-main** is $O^*(1.325^m)$. $\square$

## Data and methods

Data on somatic mutation, copy number alteration, and gene expression from 1062 cancerous tumors and 113 normal control samples (note: normal samples have only expression data) of breast tissue were downloaded from the TCGA [18]. Data of gene signatures with sizes of less than 50 genes were collected from the papers listed in Additional file 1: Table S1. A gene signature is a set of genes such that their combined expression pattern decides biological phenotype or medical condition of cells. For example, high expression of genes in a gene signature related to cell proliferation are associated with worse clinical outcomes [19, 20]. Ellsworth et al. have shown that high expression of genes in one particular gene signature are related to metastases [21]. We suppose that genes in a signature are regulated by a common signal that can be perturbed by SGA events. The perturbations of the signal may cause the abnormal expression of genes in the signature and change the behaviors of cells; thus the normal cells become cancerous ones. In this study, first, we used expression status of gene signatures as the signal readout to find tumors that had SGAs that perturbed common signals. Then, we used our new algorithm to search for cancer driver SGAs by applying mutual exclusivity only on the tumors that perturbed common signals.

Before generating the instances for the weighted mutually exclusive maximum set cover problem, we performed the following data pre-processing:

First, we found genes that had *valid* SGA events for each tumor, i.e., the SGA events would perturb the signals if those genes were on the pathways that carried the signals. If a gene had non-silent somatic mutation in a tumor, then the SGA event for the gene was valid in the tumor. If a gene had copy number alteration (with a GISTIC [22] score of 2 or $-2$) in a tumor, we further checked if the expression of the gene in the tumor had corresponding changes. For example, if a gene was amplified (with a GISTIC score 2) in a tumor, then the SGA of the gene was valid if the expression of the gene also increased significantly in the tumor. For each gene *g*, we used the distribution of the expression of the gene in all tumors with a GISTIC score 0 (no amplification and no deletion) as the background and obtained the mean $m_g$ and standard deviation $d_g$. The $m_g$ and $d_g$ were then used to make a z-transformation for the expression of the gene in tumors with copy number alteration of the gene. If the gene *g* had a GISTIC score of 2 and z-score $\ge 1.64$ or a GISTIC score of $-2$ and z-score $\le -1.64$ (the gene was up- or down-regulated with a p value of at least 0.05) in a tumor, the SGA event was valid for the gene *g* in the tumor. We removed genes that had valid SGA events in no more than five tumors and obtained a final set of genes with valid SGA events for each tumor.

Then for each gene signature, we found two tumor subsets $S_p$ and $S_n$ such that genes in the signature were expressed abnormally for all tumors in $S_p$ and had expression patterns similar to normal cells for all tumors in $S_n$. By comparing the middle value of expression of the gene in normal samples, we were able to obtain the expression changes of the gene in the tumors. We deemed that a gene was up-/down-regulated significantly in a tumor if the expression change of the gene increased/decreased at least threefold in the tumor. If more than $75\,\%$ of genes in a signature were up-regulated significantly in a tumor, we said that the gene signature was up-regulated in the tumor and added the tumor into $S_p$; similarly, we added the tumor into $S_p$ if more than $75\,\%$ of genes in the signature were down-regulated significantly (the signature was down-regulated in the tumor). A tumor was added into $S_n$ if the changes in expression level of at least $50\,\%$ of genes in the signature were less than twofold. We used a relatively strict threshold to find up-/down-regulated genes and partition tumors in order to increase the probability that the tumors in $S_p$ had SGAs that perturbed a common signal while the tumors in $S_n$ did not have SGAs that perturbed this common signal. Tumors in $S_p$ were further divided into two sub-groups according to if the gene signature was up- or down-regulated in the tumors. The sub-group sizes were usually very different, and the tumors in the smaller sub-group were removed from $S_p$ if the size of the smaller sub-group was less than $10\,\%$ of the size of the large sub-group. If the size of the small sub-group was not within $10\,\%$ of the size of the large sub-group, we did not further consider this gene signature because if the signature was up- and down-regulated in considerable cancerous tumors, it appeared less likely to be related to cancer. We continued to work on a gene signature if the $S_p$ had more than 30 tumors.

Finally, we calculated the weights of genes with SGAs for each gene signature such that the weights reflected the information between SGA events and tumors in $S_p$, which were supposed to have SGAs that perturbed a common signal. For example, if there are two genes $g_1$ and $g_2$ such that $g_1$ has 10 SGA events in tumors in $S_p$ and 2 SGA events in tumors in $S_n$ while $g_2$ has 10 SGA events in tumors in $S_p$ and 20 SGA events in tumors in $S_n$, then by intuition, $g_1$ is more likely to be on the pathway that carries the signal to regulate the expression of genes in the signature as there are too many cases where the SGA events of $g_2$ do not affect the expression of genes in the given signature. So the gene $g_1$ should have better (smaller) weight. The previous methods [5, 6] did not treat $g_1$ and $g_2$ differently. We used the hypergeometric distribution [23] to measure the information between the SGA events and tumors in $S_p$, i.e., to define the weights of genes with SGAs. The introducing of the weights is helpful for using mutual exclusivity to search for genes that are from the same pathway and carry a common signal.

After the pre-processing, then, for each gene signature, we obtained two tumor subsets, $S_p$ and $S_n$, and a weighted gene subset $G_{SGA}$, where $S_p$ was a set of tumors supposed to have SGAs that perturbed the common signal regulating the expression of genes in the signature, $S_n$ was a set of tumors not supposed to have SGAs that perturbed this common signal, and the weight of a gene in $G_{SGA}$ reflected the information of the SGA events of the gene with respect to the tumors in $S_p$ (or to the common signal). For each gene signature, we made an instance of the weighted mutually exclusive maximum set cover problem by transforming each gene *g* in $G_{SGA}$ into a subset $S_g$ of $S_p$ such that the $S_g$ was a subset of tumors that had valid SGA events for the gene *g*. The minimum, average and maximum sizes of all *X*s, $\mathcal{F}$s, and elements of $\mathcal{F}$s can be found in Additional file 1: Table S2.

For example, suppose for a given gene signature, we have $S_p = \{T_1, \ldots , T_5\}$, $G_{SGA} = \{g_1, g_2, g_3\}$, where the weights of $g_1$, $g_2$, $g_3$ are $w_1$, $w_2$, $w_3$, respectively; further, suppose that $g_1$ has valid SGAs in tumors $T_1$ and $T_2$; $g_2$ has valid SGAs in tumors $T_2$, $T_3$, and $T_4$; and $g_3$ has valid SGAs in tumors $T_2$ and $T_5$, then the instance of the weighted mutually exclusive maximum set cover problem is $X = \{T_1, \ldots , T_5\}$, $\mathcal{F} = \{\{T_1,T_2\},\{T_2,T_3,T_4\}, \{T_2,T_5\}\}$ with the weight of $\{T_1,T_2\}$, $\{T_2,T_3,T_4\}$, $\{T_2,T_5\}$ (i.e., $g_1$, $g_2$, $g_3$) to be $w_1$, $w_2$, $w_3$ respectively. Then we used our new algorithm to find the optimal solution, called the *up-stream module*, for the instance. The up-stream module is a subset of genes such that (1) the overall SGA events of the genes in the subset are mutually exclusive and cover the maximum number of tumors in $S_p$; and (2) the weight sum of genes in the subset is minimized. As for each gene signature, the SGA events of genes in the up-stream module are mutually exclusive and have strong information with respect to tumors in $S_p$, providing strong evidence that genes in the up-stream module are on the same pathway that carries the signal regulating the expression of genes in the signature.

## Results

We identified 59 up-stream modules (Additional file 1: Table S3). The sizes of the modules ranged from 3 to 12 genes, with an average of 5.66 genes. We evaluated the results by mapping them to existing knowledge using Ingenuity Pathway Analysis (IPA–http://www.ingenuity.com ); we found that 32 of our up-stream modules were significantly enriched in one of the IPA pathways (p value $<0.01$, q value $<0.02$); meanwhile 44 up-stream modules were strongly associated with different diseases (p value $<0.001$, q value $<0.001$), and among these 44, 25 of the modules were related to cancers. We understand that while knowledge about the pathways and diseases in IPA may be incomplete, it is, nonetheless, an accessible approach that has been widely used by biologists to evaluate their results. Thus, evaluations with IPA can be used as references for the overall performance of the results.

**Fig. 5:**
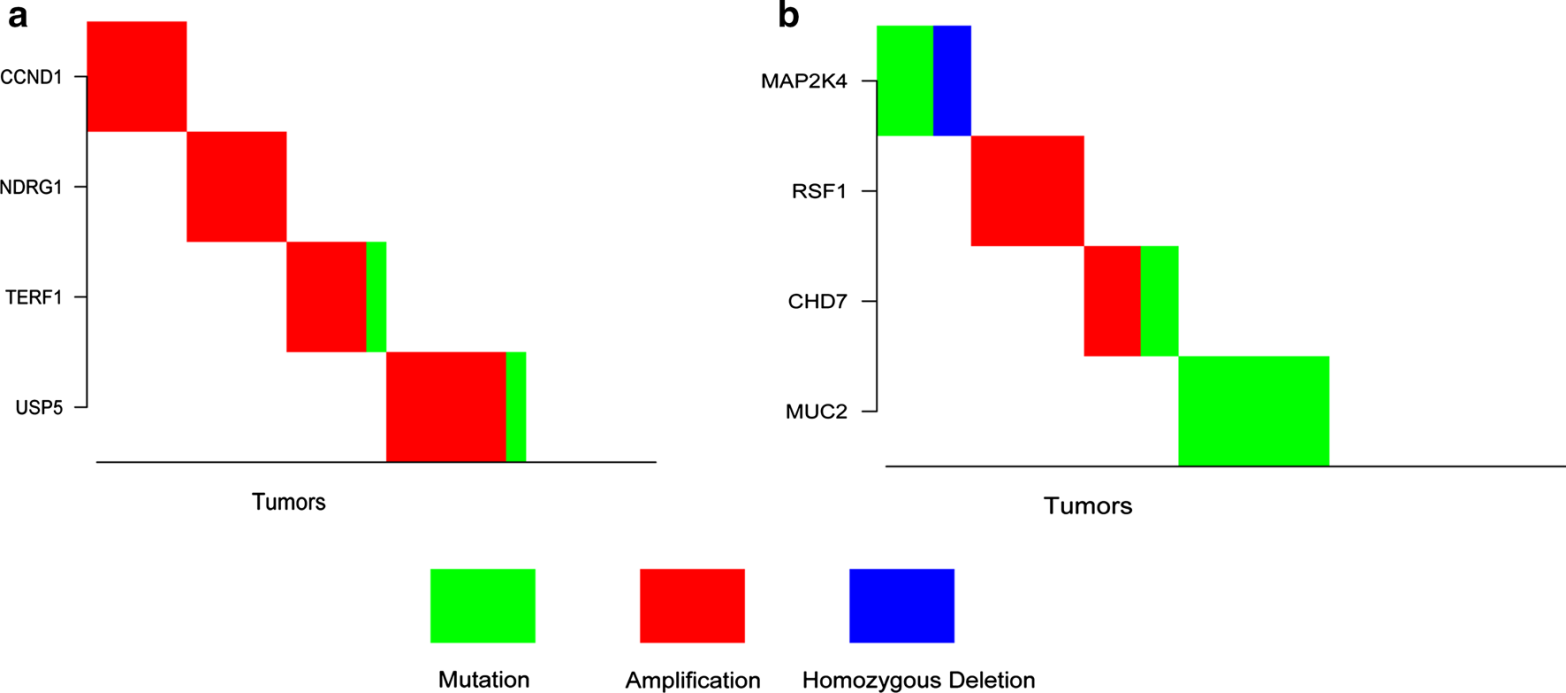
SGA events in up-stream-modules 59 and 35

We further conducted a literature search to verify the up-stream modules and found that the SGA events of genes in many modules were related to cancer or tumor development. One example is up-stream module 59, which included amplifications of CCND1 and NDRG1, and amplifications and mutations of TERF1 and USP5 (refer to Fig.5a). CCND1 is a well-known *oncogene* (the function enhancement of the gene will lead to tumors) that has been found to be amplified in breast cancer and head and neck carcinoma [24–26]. CCND1 can be used as a therapeutic target in cancer therapy [26, 27]. The amplification or overexpression of NDRG1 causes problems in hepatocellular carcinoma [28], esophageal cancer [29], and lung cancer [30]. Thus, reducing the expression of NDRG1 can decrease tumor growth rate [31]. Increased or high level TERF1/TRF1 expression has been observed in a large percentage of adult T-cell leukemia [32] and colorectal cancer samples [33]. The overexpression of USP5 has been found to be related to melanoma as it results in suppression of a very important *tumor suppressor* (the function loss of the gene will cause cancers)—p53 [34]. The above previous discoveries by other researchers support our findings for the TCGA breast cancer samples. The gene signature corresponding to up-stream module 59 was obtained from Bar-On’s paper [35]. The authors did not mention the function or biological process of this gene signature. As CCND1 in the up-stream module is a well-known gene related to the cell cycle, we presume that the genes in the signature are associated with the biological process of the cell cycle. When using the PASTAA database [36] to search for enriched transcription factor binding sites of genes in this signature, we found that the binding sites of transcription factors Ap-2alpha and Ap-2gamma were the most highly enriched region among the promoters of the genes in this signature (Additional file 1: Table S4). Transcription factors Ap-2alpha and Ap-2gamma are known to regulate genes involved in the cell cycle [37–39]. Therefore, it is highly likely that genes in this signature are involved in cell cycle processes. This presumption is further supported by additional literature, in which we found that both TRF1 [40] and USP3 [34] play a role in regulating the cell cycle. Hence we conclude that the SGA events of genes in up-stream module 59 contribute to breast tumors through the perturbation of the cell cycle.

Another example is up-stream module 35, which has four genes (refer to Fig.5b): MAP2K4, RSF1, CHD7, and MUC2; MAP2K4 had both mutations and deletions in tumors; CHD7 had amplifications and mutations; RSF1 had amplifications and MUC2 had mutations in tumors. The literature provides evidence that SGA events of genes in module 35 contribute to tumor development. MAP2K4 is highly mutated in breast tumors [41]. Ahn et al. found that MAP2K4 functioned as a tumor suppressor in lung adenocarcinoma [42]. Vang et al. discovered that ovarian cancer patients with RSF1 amplification or overexpression had a significantly worse clinical outcome [43]. Low expression of CHD7 has been associated with better survival [44], and the depletion of CHD7 has been shown to reduce cell proliferation [45]. The loss of MUC2 expression has been related to recurrence of colorectal carcinoma while the positive expression of MUC2 has been associated with longer survival [46]. The suppression of MUC2 has also been found to enhance the proliferation and invasion of colorectal cancer cells [47]. The gene signature corresponding to this up-stream module has also been related to metastasis [21]; we further checked the relations between these four genes and the biological process of metastasis. MAP2K4 was found to be a tumor and metastasis suppressor gene [42, 48], which means that mutation and deletion of MAP2K4 will enhance the metastasis of tumors. It was also found that a high expression of RSF1 was associated with lymph node metastasis in ovarian clear cell carcinoma [49]. Bajpai et al. claimed that CHD7 gain-of-function may play a role in tumor progression and that CHD7 function is essential for the expression of TWIST and SLUG genes, critical regulators of EMT and metastasis [50]. Hanski et al. found that the suppression of MUC2 was associated with liver and lymph node metastasis of colorectal carcinomas [51]. Hence, all four genes in this up-stream module are related to cell metastasis regulation, which perfectly matches the biological process of the down-stream gene signature.

Finally, we evaluated the overall performance of our method by comparing it with a well-known method, Dendrix [5], which also uses mutual exclusivity to search for driver SGAs. The goal of Dendrix is to seek solutions with a balance between coverage and mutual exclusivity of SGAs, i.e., the SGA events of genes in a solution should cover as many tumors as possible while having as few overlaps as possible. Both our method and Dendrix use the mutually exclusive property. The major advantage of our method is that each of our solutions carries the information with respect to a common signal. Dendrix has one merit in that it allows the small overlap of SGAs. Remember that for each gene signature, we obtained two tumor subsets $S_p$ and $S_n$ such that tumors in $S_p$ were supposed to have SGAs that perturbed a common signal while tumors in $S_n$ were not supposed to have SGAs that perturbed this common signal. Hence, if genes in a solution have any SGA events that perturb the common signal, the SGA events should occur mostly or be enriched in tumors in $S_p$ and happen only in a small number of tumors that are assigned into $S_n$ by computation or data error in the last step. We used the hypergeometric distribution to measure the enrichment p-values of SGAs, where p values were associated with the information of the SGA events of a solution with respect to a common signal.

**Fig. 6:**
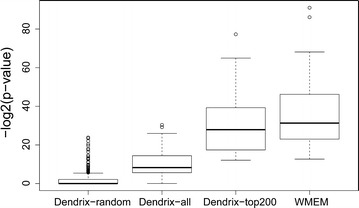
Comparing the enrichment p values of different methods

There are three major innovations in our framework. First, we applied mutual exclusivity to a preselected subset $S_p$ of tumors that are supposed to have SGA events that perturb a common signal that regulates the expression of a given gene signature. Second, we defined weights for SGA genes, or pathway candidates, which reflect the information with respect to the common signal that regulates the gene signature and pre-selected candidates of top weights for finding driver SGAs/signaling pathways related to cancer development. Finally, we presented an exact algorithm that guarantees to find the optimal solution of the model to search for driver SGAs. We evaluated the performance of our method according to the above mentioned three aspects. A comparison of results from different methods or settings is shown in the Fig. 6, where the Y axis represents the negative log (base 2) values of the enrichment p values. First we applied Dendrix to $S_p$s that are subsets of tumors decided by gene signatures (Dendrix-all) and $S_p'$s that are random tumor subsets with the same sizes as corresponding $S_p$s (Dendrix-random). Dendrix-random is similar to the situation in the original Dendrix, as all tumors in a given data set can be considered as a random tumor subset from all cancer tumors. The figure shows that using preselected tumor subsets improves the results by an average of 8.62. Then we applied Dendrix to $S_p$s and constrained SGA gene candidates to those with weights within the top 200 and less than 0.2 (Dendrix-top200). The results were then further improved more than 20.12 on average. Finally, we applied our own exact algorithm (WMEM) to the same data set as that used for the Dendrix-top200. The results were improved another 6.59 on average. As the values in the figure were log (base 2) values of the p-values, a difference of 6.59 corresponded to a 96-fold difference in the original p-values. Therefore, by considering and using the weights of SGAs, we can obtain solutions with high information with respect to common signals.

## Conclusion and future works

In this paper, we introduced an innovative model and an efficient exact algorithm that can handle both mutual exclusivity and the weight arising from it. The new model, which is signal-based and considers the information with respect to the common signals, utilizes mutual exclusivity in a proper manner while the new exact algorithm guarantees to obtain optimal solutions of the new method. The results showed that our new method and algorithm could discover the true causes behind the phenotypes, such as what SGA events lead to abnormality of the cell cycle or make the cell metastasis lose control in tumors; thus, it provides target candidates for precision (or target) therapeutics.

The weighted mutually exclusive maximum set cover problem for the new model is NP-hard. The main reason the NP-hard problem is handled so efficiently by our new algorithm is that we have utilized parameterized technologies. This means that the running time of the new algorithm increases exponentially only in the parameter, which can be constrained within a small number, rather than to the problem input size, which is usually big and difficult to control. The running time of our algorithm is $O^*(1.325^m)$, which can solve the NP-hard problems of application in a reasonable amount of time. We believe that the running time can be further improved.

While reducing the running time to solve the weighted mutually exclusive maximum set cover problem, which has important applications in cancer study, is a benefit, another variant of the problem should be particularly paid attention to. Strict mutual exclusivity is the extreme case; some tumors may have more than one SGA event to perturb one particular pathway. Hence, we need to relax the strict mutual exclusivity and modify the problem to a weighted small overlapped maximum set cover problem, which allows each tumor to be covered by a small number (such as two or three) of SGA events. This is another important problem that is worthwhile and needs to be solved quickly.

## Additional file


10.1186/s13015-016-0073-9 Supplementary materials.

## Footnotes

1 **Note:** We use a star * to represent that the polynomial part of the time complexity is neglected.
