# Supplementary material for: An exact algorithm for finding cancer driver somatic genome alterations: the weighted mutually exclusive maximum set cover problem
Source: Algorithms Mol Biol. 2016 May 4;11:11. doi: 10.1186/s13015-016-0073-9 (PMC4855522; doi:10.1186/s13015-016-0073-9)
Supplement: Supplementary file 1 — 10.1186/s13015-016-0073-9 Supplementary materials. [file 13015_2016_73_MOESM1_ESM.pdf]

Table S1: Sources of 59 gene signatures.

Signature ID	Reference		Signature ID	Reference
1	[1]--Table3a-1		31	[2]--SuppTable7
2	[3]--Table1c		32	[2]--SuppTable8
3	[4]--Table4		33	[5]--Table4
4	[6]--Table2b		34	[5]--Table5
5	[7]--TableII		35	[8]--Table2b
6	[9]--Table4		36	[10]--TableS1
7	[11]--Table2		37	[10]--TableS2
8	[12]--Table2		38	[10]--TableS3
9	[13]--Table2		39	[14]--Table3
10	[15]--Table5		40	[16]--SuppTable2b
11	[17]--Table4		41	[16]--SuppTable6
12	[18]--Table1b		42	[19]--Table2
13	[20]--Table1		43	[21]--TableS3a
14	[22]--Figure1B		44	[23]--Table2a
15	[24]--Table2		45	[23]--Table2b
16	[25]--SuppTable2		46	[26]--SuppTable2
17	[27]--Table5		47	[28]--SuppTable5
18	[29]--Table2b		48	[30]--Table1
19	[31]--Table1a		49	[32]--Figure1a
20	[33]--Table1		50	[34]--Table1
21	[35]--Table2		51	[36]--Table2
22	[37]--TableS3		52	[38]--Table3
23	[39]--Table4		53	[40]--Table2
24	[41]--Table3		54	[42]--SuppTable9
25	[43]--SuppTable3B		55	[44]--Table4a
26	[45]--SuppFigure2a		56	[46]--Table1
27	[47]--TableS3a		57	[48]--Table1
28	[49]--TableA10		58	[50]--Table2a
29	[2]--SuppTable4		59	[51]--TableS2
30	[2]--SuppTable6			

Table S2: The minimum, average and maximum sizes of Xs, $\mathcal{F}$s, and elements of $\mathcal{F}$s

	Min size	Avg. size	Max size
$\|\mathcal{F}\|$	44	158.5423729	200
|X|	23	75.77966102	481
|f|	5	9.777955955	119

Note: |f| represents the sizes of elements in all $\mathcal{F}$s.

Table S3: All 59 up-stream modules

Up-stream ID	Genes
1	EZR; CALB1; KIAA1109; GSDMB; SAGE1;
2	SST; ZIC5; ATP1B4; AZIN1;
	GSDMB; C1orf85; LRIG1; SLURP1;
4	ZNF701; FOLR4; PPP1R16A; DUSP16; CCDC57; SNORD44; SNORD47; SNORD74; SNORD75; SNORD77; SNORD78; SNORD80; SNORD81;
5	CSMD2; OR2AT4; PARG; ZNF517; KDM2A; MRPL45;
6	A2ML1; RAB13; DOCK9; CACNA1C; SCN8A; PDCD6;
7	RAI1; HEXIM1; ZNF704; MFHAS1; ACAD10; RBM38; CABP2;
8	CEACAM18; SLCO5A1; COPS7A; SGK196; UBFD1; RNF114;
9	ANK1; SDK2; N4BP2L2; C8orf47; YEATS4; SSR2;
10	NCOA3; KPNA2; ST3GAL1; THAP1; PAK1;
11	ATN1; SPTA1; DDI1; ST3GAL1;
12	TNFRSF10A; CACNA1H; TRPS1; KCNG4; PARD6B; SGK494; KLHDC9; BAGE2; MUC2;
13	RABIF; ZHX2; YEATS4;
14	HELZ; N4BP2L2; MUC12; FNTA; SLCO1B3;
15	APH1A; MUC2; SAP30BP;
16	FMOD; AQP11; FOXA1; MLL; PITPNC1; MUC5B; THAP1; GDPD1;
17	TACO1; NARS2; ZNF703; RFWD2;
18	LOC728743; SLU7; GATA3; MFHAS1; VDAC3;
19	RB1; ZNF703; ESRP1;
20	SLC7A6OS; PPP2CA; TRIM58; RBM4B; R3HDM2; IPP; ABHD12; MPDU1; FRS2; RP1L1;
21	VDAC3; USPL1; HGS; TROVE2;
22	C1orf74; KIFC2; NME2; SLC37A2; TMEM74;
23	PTTG1; USP35; SPATA17; DLGAP4; MUC5B; CHD1L;
24	RBM4B; GABRA6; TSPAN9; MGMT; NAA25; LOC728743; CLDN23; USP15; PDE8A; PSKH2; PPP1R16A;
25	MAP3K1; FOLR4; ANK1; SNORD44; SNORD47; SNORD74; SNORD75; SNORD77; SNORD78; SNORD80; SNORD81; PPM1D;
26	FRG1B; PMF1; VCPIP1;
27	PRKRIR; MUC4; TANC2;
28	FLT3; SET; FAM63A; ZNF2; SPCS2; C16orf52; AFF3; SLC25A37; SUV420H1;
29	C1orf61; LARP1; CARNS1; ESPNP; GRID2; ZNF704; USP35;
30	FAM63A; TOX4; ESRP1;
31	TMEM134; EIF2AK1; PARP15; PSCA; CALU; TSPAN14; CACNG1; DCTN5; P2RY2; MUC2;
32	AKT2; C17orf58; HMBOX1; NADSYN1; TRPM3; LACTB2;
33	LRBA; ARHGEF10; APOA1BP;
34	MUC12; ZNF703; USP34; TRHR; SUPT5H;
35	MAP2K4; RSF1; CHD7; MUC2;

Table S3 continue

36	TFDP1; FBXL14; FAM189B; AP3M2;
37	TFDP1; FBXL14; FAM189B; AP3M2;
38	TFDP1; FBXL14; FAM189B; AP3M2;
39	NARS2; HGS; AGPAT6; TOMM20;
40	APOBEC4; SCAND2; FOXA1; CTRB2; PRR14; TEX2; ADAM18;
41	MAPK7; EI24; DDX5; PTTG1; RIPK2;
42	RPRD2; USP36; DDHD2;
43	CNGB3; GATA3; PCMTD2; TOR3A; RYR1;
44	SERPINI2; PARD6B; MTX1; UPB1; MBD4; NDUFS5; LOC442421; CBX4; THAP1;
45	SERPINI2; PARD6B; MTX1; UPB1; MBD4; NDUFS5; LOC442421; CBX4; THAP1;
46	GBAP1; THAP1; RECQL5; MUC5B;
47	UVRAG; TMEM186; RIMS2; USF1; MLL3; HDAC5;
48	SI; TG; OR10Z1;
49	N4BP2L1; CHD4; GPATCH2; SMG5; TP53INP1;
50	SEPT10; ADAM15; CDKN2B; ANKRD46;
51	MUC2; EEF2K; ABCA5; FCGBP; SDK2; POLB; GPATCH4;
52	ZNF695; DDX11; CCND1; DSCC1;
53	OR8D2; LOC728989; KIAA1324; SH3PXD2A; AKTIP; ADAMTSL1; SLC24A1; ABCC11; MGAM; METTL2A;
54	NFKBIB; TRPC5; COL1A2; RNMT; LPHN2; LYN; ZBTB2; THBS4; SLK; CD244; SALL1; EMG1;
55	TBL3; GDI1; CIB1; NR5A2; CTCF; CDRT15; AGAP7; C8orf86; SHANK2;
56	MRPL30; ZBTB43; SNORA8; POLR1E; PIK3CA;
57	SLC25A37; NUP93; PTBP1; CCNI; ERCC4; PSCA; POP4; LCT; WDR53;
58	PKHD1L1; RYR2; PLEKHF1; CUL3; AARS2;
59	CCND1; NDRG1; TERF1; USP5;

Table S4: Use PASTTA to find possible transcription factors regulate genes in the gene signature 59.

Rank	Matrix	Transcription Factor	Association Score	P-Value
1	AP2_Q6	Ap-2 , Ap-2alpha	3.123	0.00e+00
2	AP2_Q3	Ap-2alpha , Ap-2alphaa	2.814	9.55e-04
3	AP2ALPHA_01	Ap-2alpha , Ap-2alphaa	2.641	1.88e-03
4	AP2GAMMA_01	Ap-2gamma	2.641	1.88e-03
5	AR_02	Ar	2.521	2.86e-03
6	PR_02	N/A	2.521	2.86e-03
7	NFKAPPAB50_01	N/A	2.385	3.82e-03
8	AP2_Q6_01	Ap-2 , Ap-2alpha	2.123	7.54e-03
9	STAF_01	Staf	2.123	7.54e-03
10	XPF1_Q6	N/A	2.044	7.54e-03
11	MIF1_01	N/A	1.868	1.32e-02
12	STAT3_01	Stat3	1.868	1.32e-02
13	AR_Q2	Ar	1.819	1.51e-02
14	GR_01	N/A	1.743	1.70e-02
15	PR_01	N/A	1.743	1.70e-02
16	AR_03	Ar	1.618	2.27e-02
17	GC_01	N/A	1.618	2.27e-02
18	STAT_Q6	Stat1alpha , Stat1beta	1.567	2.63e-02
19	STAF_02	Staf	1.567	2.63e-02

1. Iizaka M, Furukawa Y, Tsunoda T, Akashi H, Ogawa M, et al. (2002) Expression profile analysis of colon cancer cells in response to sulindac or aspirin. Biochem Biophys Res Commun 292: 498-512.
2. Andreeff M, Ruvolo V, Gadgil S, Zeng C, Coombes K, et al. (2008) HOX expression patterns identify a common signature for favorable AML. Leukemia 22: 2041-2047.
3. Bigler D, Gulding KM, Dann R, Sheabar FZ, Conaway MR, et al. (2003) Gene profiling and promoter reporter assays: novel tools for comparing the biological effects of botanical extracts on human prostate cancer cells and understanding their mechanisms of action. Oncogene 22: 1261-1272.
4. Weston G, Trajstman AC, Gargett CE, Manuelpillai U, Vollenhoven BJ, et al. (2003) Fibroids display an anti-angiogenic gene expression profile when compared with adjacent myometrium. Mol Hum Reprod 9: 541-549.
5. Bhattacharya S, Srisuma S, Demeo DL, Shapiro SD, Bueno R, et al. (2009) Molecular biomarkers for quantitative and discrete COPD phenotypes. Am J Respir Cell Mol Biol 40: 359-367.
6. Qiu J, Gunaratne P, Peterson LE, Khurana D, Walsham N, et al. (2003) Novel potential ALL low-risk markers revealed by gene expression profiling with new high-throughput SSH-CCS-PCR. Leukemia 17: 1891-1900.
7. Maehara S, Tanaka S, Shimada M, Shirabe K, Saito Y, et al. (2004) Selenoprotein P, as a predictor for evaluating gemcitabine resistance in human pancreatic cancer cells. Int J Cancer 112: 184-189.
8. Ellsworth RE, Seebach J, Field LA, Heckman C, Kane J, et al. (2009) A gene expression signature that defines breast cancer metastases. Clin Exp Metastasis 26: 205-213.
9. Williams SS, Mear JP, Liang HC, Potter SS, Aronow BJ, et al. (2004) Large-scale reprogramming of cranial neural crest gene expression by retinoic acid exposure. Physiol Genomics 19: 184-197.

10. Cavarra E, Fardin P, Fineschi S, Ricciardi A, De Cunto G, et al. (2009) Early response of gene clusters is associated with mouse lung resistance or sensitivity to cigarette smoke. Am J Physiol Lung Cell Mol Physiol 296: L418-429.
11. Gatalica Z, Velagaleti G, Kuivaniemi H, Tromp G, Palazzo J, et al. (2005) Gene expression profile of an adenomyoepithelioma of the breast with a reciprocal translocation involving chromosomes 8 and 16. Cancer Genet Cytogenet 156: 14-22.
12. Mahadevan B, Keshava C, Musafia-Jeknic T, Pecaj A, Weston A, et al. (2005) Altered gene expression patterns in MCF-7 cells induced by the urban dust particulate complex mixture standard reference material 1649a. Cancer Res 65: 1251-1258.
13. Roth A, Baerlocher GM, Schertzer M, Chavez E, Duhrsen U, et al. (2005) Telomere loss, senescence, and genetic instability in CD4+ T lymphocytes overexpressing hTERT. Blood 106: 43-50.
14. Protiva P, Cross HS, Hopkins ME, Kallay E, Bises G, et al. (2009) Chemoprevention of colorectal neoplasia by estrogen: potential role of vitamin D activity. Cancer Prev Res (Phila) 2: 43-51.
15. Kalajzic I, Staal A, Yang WP, Wu Y, Johnson SE, et al. (2005) Expression profile of osteoblast lineage at defined stages of differentiation. J Biol Chem 280: 24618-24626.
16. Rangasamy T, Misra V, Zhen L, Tankersley CG, Tuder RM, et al. (2009) Cigarette smoke-induced emphysema in A/J mice is associated with pulmonary oxidative stress, apoptosis of lung cells, and global alterations in gene expression. Am J Physiol Lung Cell Mol Physiol 296: L888-900.
17. Oostendorp RA, Robin C, Steinhoff C, Marz S, Brauer R, et al. (2005) Long-term maintenance of hematopoietic stem cells does not require contact with embryo-derived stromal cells in cocultures. Stem Cells 23: 842-851.
18. Inamura K, Fujiwara T, Hoshida Y, Isagawa T, Jones MH, et al. (2005) Two subclasses of lung squamous cell carcinoma with different gene expression profiles and prognosis identified by hierarchical clustering and non-negative matrix factorization. Oncogene 24: 7105-7113.
19. Price KL, Woolf AS, Long DA (2009) Unraveling the genetic landscape of bladder development in mice. J Urol 181: 2366-2374.
20. Oswald J, Steudel C, Salchert K, Joergensen B, Thiede C, et al. (2006) Gene-expression profiling of CD34+ hematopoietic cells expanded in a collagen I matrix. Stem Cells 24: 494-500.
21. Bonuccelli G, Casimiro MC, Sotgia F, Wang C, Liu M, et al. (2009) Caveolin-1 (P132L), a common breast cancer mutation, confers mammary cell invasiveness and defines a novel stem cell/metastasis-associated gene signature. Am J Pathol 174: 1650-1662.
22. Hernandez-Gonzalez I, Gonzalez-Robayna I, Shimada M, Wayne CM, Ochsner SA, et al. (2006) Gene expression profiles of cumulus cell oocyte complexes during ovulation reveal cumulus cells express neuronal and immune-related genes: does this expand their role in the ovulation process? Mol Endocrinol 20: 1300-1321.
23. Yoshihara K, Tajima A, Komata D, Yamamoto T, Kodama S, et al. (2009) Gene expression profiling of advanced-stage serous ovarian cancers distinguishes novel subclasses and implicates ZEB2 in tumor progression and prognosis. Cancer Sci 100: 1421-1428.
24. Jorgensen JR, Juliusson B, Henriksen KF, Hansen C, Knudsen S, et al. (2006) Identification of novel genes regulated in the developing human ventral mesencephalon. Exp Neurol 198: 427-437.
25. Doane AS, Danso M, Lal P, Donaton M, Zhang L, et al. (2006) An estrogen receptor-negative breast cancer subset characterized by a hormonally regulated transcriptional program and response to androgen. Oncogene 25: 3994-4008.
26. Chambers KF, Bacon JR, Kemsley EK, Mills RD, Ball RY, et al. (2009) Gene expression profile of primary prostate epithelial and stromal cells in response to sulforaphane or iberin exposure. Prostate 69: 1411-1421.
27. Gate L, Langlais C, Micillino JC, Nunge H, Bottin MC, et al. (2006) Bitumen fume-induced gene expression profile in rat lung. Toxicol Appl Pharmacol 215: 83-92.

28. Zangrando A, Dell'orto MC, Te Kronnie G, Basso G (2009) MLL rearrangements in pediatric acute lymphoblastic and myeloblastic leukemias: MLL specific and lineage specific signatures. BMC Med Genomics 2: 36.
29. West AN, Neale GA, Pounds S, Figueredo BC, Rodriguez Galindo C, et al. (2007) Gene expression profiling of childhood adrenocortical tumors. Cancer Res 67: 600-608.
30. Marella NV, Malyavantham KS, Wang J, Matsui S, Liang P, et al. (2009) Cytogenetic and cDNA microarray expression analysis of MCF10 human breast cancer progression cell lines. Cancer Res 69: 5946-5953.
31. Mahadevan D, Cooke L, Riley C, Swart R, Simons B, et al. (2007) A novel tyrosine kinase switch is a mechanism of imatinib resistance in gastrointestinal stromal tumors. Oncogene 26: 3909-3919.
32. Ibanez de Caceres I, Cortes-Sempere M, Moratilla C, Machado-Pinilla R, Rodriguez-Fanjul V, et al. (2010) IGFBP-3 hypermethylation-derived deficiency mediates cisplatin resistance in non-small-cell lung cancer. Oncogene 29: 1681-1690.
33. Nakayama R, Nemoto T, Takahashi H, Ohta T, Kawai A, et al. (2007) Gene expression analysis of soft tissue sarcomas: characterization and reclassification of malignant fibrous histiocytoma. Mod Pathol 20: 749-759.
34. Utispan K, Thuwajit P, Abiko Y, Charngkaew K, Paupairoj A, et al. (2010) Gene expression profiling of cholangiocarcinoma-derived fibroblast reveals alterations related to tumor progression and indicates periostin as a poor prognostic marker. Mol Cancer 9: 13.
35. Uchimura S, Iizuka N, Tamesa T, Miyamoto T, Hamamoto Y, et al. (2007) Resampling based on geographic patterns of hepatitis virus infection reveals a common gene signature for early intrahepatic recurrence of hepatocellular carcinoma. Anticancer Res 27: 3323-3330.
36. Cleaver AL, Beesley AH, Firth MJ, Sturges NC, O'Leary RA, et al. (2010) Gene-based outcome prediction in multiple cohorts of pediatric T-cell acute lymphoblastic leukemia: a Children's Oncology Group study. Mol Cancer 9: 105.
37. Yu J, Yu J, Rhodes DR, Tomlins SA, Cao X, et al. (2007) A polycomb repression signature in metastatic prostate cancer predicts cancer outcome. Cancer Res 67: 10657-10663.
38. Li M, Zhao ZW, Zhang Y, Xin Y (2011) Over-expression of Ephb4 is associated with carcinogenesis of gastric cancer. Dig Dis Sci 56: 698-706.
39. Wlodarski MW, Nearman Z, Jankowska A, Babel N, Powers J, et al. (2008) Phenotypic differences between healthy effector CTL and leukemic LGL cells support the notion of antigen-triggered clonal transformation in T-LGL leukemia. J Leukoc Biol 83: 589-601.
40. Zhu H, Zhao H, Collins CD, Eckenrode SE, Run Q, et al. (2003) Gene expression associated with interferon alfa antiviral activity in an HCV replicon cell line. Hepatology 37: 1180-1188.
41. Zafrakas M, Tarlatzis BC, Streichert T, Pournaropoulos F, Wolfle U, et al. (2008) Genome-wide microarray gene expression, array-CGH analysis, and telomerase activity in advanced ovarian endometriosis: a high degree of differentiation rather than malignant potential. Int J Mol Med 21: 335-344.
42. Thimmulappa RK, Lee H, Rangasamy T, Reddy SP, Yamamoto M, et al. (2006) Nrf2 is a critical regulator of the innate immune response and survival during experimental sepsis. J Clin Invest 116: 984-995.
43. Harvell DM, Richer JK, Singh M, Spoelstra N, Finlayson C, et al. (2008) Estrogen regulated gene expression in response to neoadjuvant endocrine therapy of breast cancers: tamoxifen agonist effects dominate in the presence of an aromatase inhibitor. Breast Cancer Res Treat 112: 489-501.
44. Miller WR, Larionov AA, Renshaw L, Anderson TJ, White S, et al. (2007) Changes in breast cancer transcriptional profiles after treatment with the aromatase inhibitor, letrozole. Pharmacogenet Genomics 17: 813-826.

45. Vicent S, Luis-Ravelo D, Anton I, Garcia-Tunon I, Borras-Cuesta F, et al. (2008) A novel lung cancer signature mediates metastatic bone colonization by a dual mechanism. Cancer Res 68: 2275-2285.
46. Tan Q, Thomassen M, Kruse TA (2007) Feature selection for predicting tumor metastases in microarray experiments using paired design. Cancer Inform 3: 213-218.
47. Haug JS, He XC, Grindley JC, Wunderlich JP, Gaudenz K, et al. (2008) N-cadherin expression level distinguishes reserved versus primed states of hematopoietic stem cells. Cell Stem Cell 2: 367-379.
48. Ueno S, Tatetsu H, Hata H, Iino T, Niiro H, et al. (2009) PU.1 induces apoptosis in myeloma cells through direct transactivation of TRAIL. Oncogene 28: 4116-4125.
49. Murat A, Migliavacca E, Gorlia T, Lambiv WL, Shay T, et al. (2008) Stem cell-related "self-renewal" signature and high epidermal growth factor receptor expression associated with resistance to concomitant chemoradiotherapy in glioblastoma. J Clin Oncol 26: 3015-3024.
50. Van Loo P, Tousseyn T, Vanhentenrijk V, Dierickx D, Malecka A, et al. (2010) T-cell/histiocyte-rich large B-cell lymphoma shows transcriptional features suggestive of a tolerogenic host immune response. Haematologica 95: 440-448.
51. Bar-On L, Birnberg T, Lewis KL, Edelson BT, Bruder D, et al. (2010) CX3CR1+ CD8alpha+ dendritic cells are a steady-state population related to plasmacytoid dendritic cells. Proc Natl Acad Sci U S A 107: 14745-14750.
